# Frailty predicts recurrence after laparoscopic Nissen fundoplication with mesh cruroplasty for giant sliding hiatal hernia with severe reflux esophagitis in elderly patients: a multicenter retrospective study

**DOI:** 10.1007/s10029-025-03416-6

**Published:** 2025-07-18

**Authors:** Tamer. A. A. M. Habeeb, Abdulzahra Hussain, Alberto Aiolfi, Jose Bueno- Lledó, Massimo Chiaretti, Igor A. Kryvoruchko, Mohammad Kermansaravi, Abdelrahman Nimeri, Abd Al-Kareem Elias, Saad Mohamed Ali Ahmed, Esmail Tharwat Kamel Awad, Mohamed. A. Gadallah, Ahmed Khyrallh, Mohammed H. Alsayed, Mohamed Fathy Labib, Sobhy Rezk Ahmed Teama, Abdelhafez Seleem, Mohammed Hassan Elshafey, Mostafa Mahmoud Salama Mostafa, Hamdi Elbelkasi, Mahmoud Ali Abou Zaid, Ahmed Hamdy, Mohamed Ibrahim Abo Alsaad, Maged Z. Youssef, Rasha Mohamed Motawea Ali, Ibtsam AbdelMaksoud Mohamed El Shamy, Ahmed Salah Arafa, Ibrahim A. Heggy, Sameh Mohamed Naguib, Tamer Wasefy, Mohamed Abozaid, Tamer Mohamed Elshahidy, Abdelshafy Mostafa, Mohamed Elnemr, Abdelrahman Mohamed Hasanin Nawar, Mostafa M. Khairy, Ahmed Mesbah Abdelaziz, Abdelfatah H. Abdelwanis, Ahmed M. El Teliti

**Affiliations:** 1 Department of General Surgery, Faculty of Medicine, Zagazig University, 1 Faculty of Medicine Street, Zagazig, Sharkia Egypt; 2 General Surgery Department, Homerton University Hospital, London, UK; 3 University of Alkafeel, Alkafeel, Iraq; 4 Division of General Surgery, Department of Biomedical Science for Health, I.R.C.C.S. Ospedale Galeazzi-Sant’Ambrogio, University of Milan, Milan, Italy; 5 Hospital Universitari i Politècnic la Fe, Valencia, Spain; 6 Department of General Surgery Specialties and Organ Transplant, Faculty of Pharmacy and Medicine, Sapienza Rome University, Rome, Italy; 7 Department of Surgery, No.2, Kharkiv National Medical University, kharkivs’Ka oblast, Ukraine; 8 Department of Surgery, Division of Minimally Invasive and Bariatric Surgery, Minimally Invasive Surgery Research Center, Hazrat-E Fateme Hospital, School of Medicine, Iran University of Medical Sciences, Tehran, Iran; 9 Department of Surgery, Center for Metabolic and Bariatric Surgery, Brigham and Women’s Hospital, Harvard Medical School, Boston, MA USA; 10 Department of General Surgery, Faculty of Medicine, Al-Azhar University, Assuit Branch, Assuit, Egypt; 11 General Surgery Department, Faculty of Medicine, Al-Azhar University, Cairo, Egypt; 12 Mataryia Teaching Hospital (GOTHI), Cairo, Egypt; 13 General Surgery Department, El Mahala Hepatic Institute, Al Gharbia, Egypt; 14 Department of Hepato-Bilio-Pancreatic (HBP) Surgery, National Hepatology and Tropical Medicine Research Institute, Cairo, Egypt; 15 General Surgery Department-Faculty of Medicine, Merit University, Sohag, Egypt; 16 Lecturer of General Surgery, Faculty of Medicine, October 6th University, Giza Governorate, Egypt; 17 Department of General Surgery, Faculty of Medicine for Girls, Al Azhar University, Cairo, Egypt

**Keywords:** Elderly, Frailty, Gastroesophageal reflux disease, Giant HH, Mesh cruroplasty, Nissen fundoplication

## Abstract

**Purpose:**

Giant sliding hiatal hernias (HH) are prevalent in the elderly population (EP) and often present with multiple comorbidities and a high surgical risk. Frailty has been increasingly recognized as a predictor of surgical outcomes in the EP. This study assessed the rate of recurrent sliding HH following *mesh cruroplasty* and laparoscopic Nissen fundoplication (LNF), and evaluated frailty as a potential risk factor of recurrence.

**Methods:**

This retrospective multicenter study included 266 patients aged ≥ 60 years who underwent *mesh cruroplasty and* LNF for giant sliding HH (> 5 cm) *with severe reflux esophagitis (Demeester score > 100)* between March 2016 and March 2022, stratified into non-recurrence (*n* = 241) and recurrence (*n* = 25) HH.

**Results:**

The mean age was 66.92 ± 4.3 years vs. 67.79 ± 3.7 years in the non-recurrence and recurrence group, respectively. Twenty-five (9.4%) patients developed recurrent HH, with a median size of 5.2 cm (4.1–6.0 cm), and the median time from surgery to recurrence was 16 months (13–20 months). Frailty was significantly correlated with recurrence, with moderately and severely frail patients demonstrating higher recurrence rates (44% vs. 17%, *p* = 0.02). Multivariate analysis confirmed that frailty was an independent predictor of recurrence (odds ratio [OR], 1.4; 95% CI, 1.003–1.982; *p* = 0.04). Time to recurrence included mild frailty (75% recurrence rate within 16 months), moderate frailty (90.9% recurrence within 12 months), and severe frailty (80% recurrence within 9 months).

**Conclusions:**

Frailty was an independent predictor of HH recurrence. Integrating frailty assessment into preoperative workflows could optimize patient selection and outcomes.

**Supplementary Information:**

The online version contains supplementary material available at 10.1007/s10029-025-03416-6.

## Introduction

The rising prevalence of sliding hiatal hernia (HH) parallels global demographic aging, affects is 24.2% of the elderly population (EP), with 33.3% concurrent GERD [1–4]. Elderly patients undergoing surgical repair of giant HH demonstrate significantly higher complication rates than younger patients, primarily attributable to their greater burden of comorbidities [5], while some studies have reported comparable surgical outcomes [6].

The primary indication for surgical intervention in type I HH is refractory GERD symptoms following failed medical management [7]. Standard surgical approaches include Nissen or Toupet fundoplication to augment the lower esophageal sphincter mechanism [8], typically combined with cruroplasty to reduce the hiatal aperture [9]. Although pure suture cruroplasty demonstrates a recurrence rate approaching 59% at 5-year follow-up [10], the role of mesh reinforcement remains debated [11]. The current guidelines acknowledge potential recurrence reduction with mesh augmentation but caution against routine use due to insufficient evidence and risks of mesh-related complications, including dysphagia and esophageal erosion [12]. Selective mesh application may be considered in cases of substantial hiatal defects or compromised crural integrity [13], with some studies demonstrating reduced recurrence and intrathoracic migration rates [13]. Recent proposals advocate for patient-specific algorithms that incorporate hiatal dimensions, crural quality, and recurrence history [10, 13], although the absence of high-quality evidence precludes definitive recommendations regarding routine mesh reinforcement in type I HH repairs.

Despite the optimization of surgical techniques, hiatal hernia repair carries a risk of hernia recurrence (12–16% radiologic, 3.2–11% clinical), which may require reoperation, potentially adversely affecting long-term patient satisfaction and quality of life. HH recurrence is influenced by various factors, including surgeon experience, inadequate short esophagus, mesh incorporation, postoperative stressors, and patient factors, such as obesity [14–16]. Symptomatic recurrences necessitating reoperation pose greater operative difficulty due to scar tissue formation and altered anatomy, resulting in higher perioperative morbidity rates than primary repairs [17].

Frailty is a clinical syndrome characterized by diminished physiological reserves and increased vulnerability to stressors, which is distinct from chronological aging. Frailty quantifies an individual’s biological resilience, particularly in geriatric populations [18]. In an era of population aging, in which patients aged ≥ 60 years now account for more than 50% of all surgical procedures, frailty assessment has become clinically essential for predicting postoperative outcomes [19]. While chronological age alone should not determine surgical candidacy, age-specific considerations remain critical in clinical decision making. In patients aged ≥ 60 years, a comprehensive geriatric assessment provides an evidence-based framework for evaluating whether the potential benefits of surgery justify the associated risks and often supersedes the traditional outcome metrics [20]. A recent study demonstrated that frailty is a significant predictor of perioperative complications [21]. An analysis of the National Surgical Quality Improvement Program (NSQIP) database revealed that physiological frailty, rather than chronological age, independently predicted postoperative complications. This finding underscores the importance of frailty assessment over age-based risk stratification in surgical decision-making [22]. Oor et al. demonstrated that rigorous patient selection incorporating frailty assessments and functional status evaluations yielded favourable postoperative outcomes independent of chronological age. Their multicenter analysis revealed comparable complication rates between carefully selected octogenarians and younger cohorts, thereby supporting age-neutral selection criteria when a comprehensive preoperative assessment is employed [23].

Giant sliding HH in EP demonstrates elevated recurrence rates attributable to multiple factors. Although the impact of frailty on tissue healing and resilience is biologically plausible, the role of frailty in HH recurrence remains unclear. Notably, no previous study has systematically evaluated frailty as a risk factor for recurrence following giant sliding HH repair in patients with EP. The present study hypothesized that frailty independently predicts recurrence in patients aged ≥ 60 years who underwent laparoscopic giant sliding HH repair.

## Methods

### Study design and eligibility criteria

**Fig. 1:**
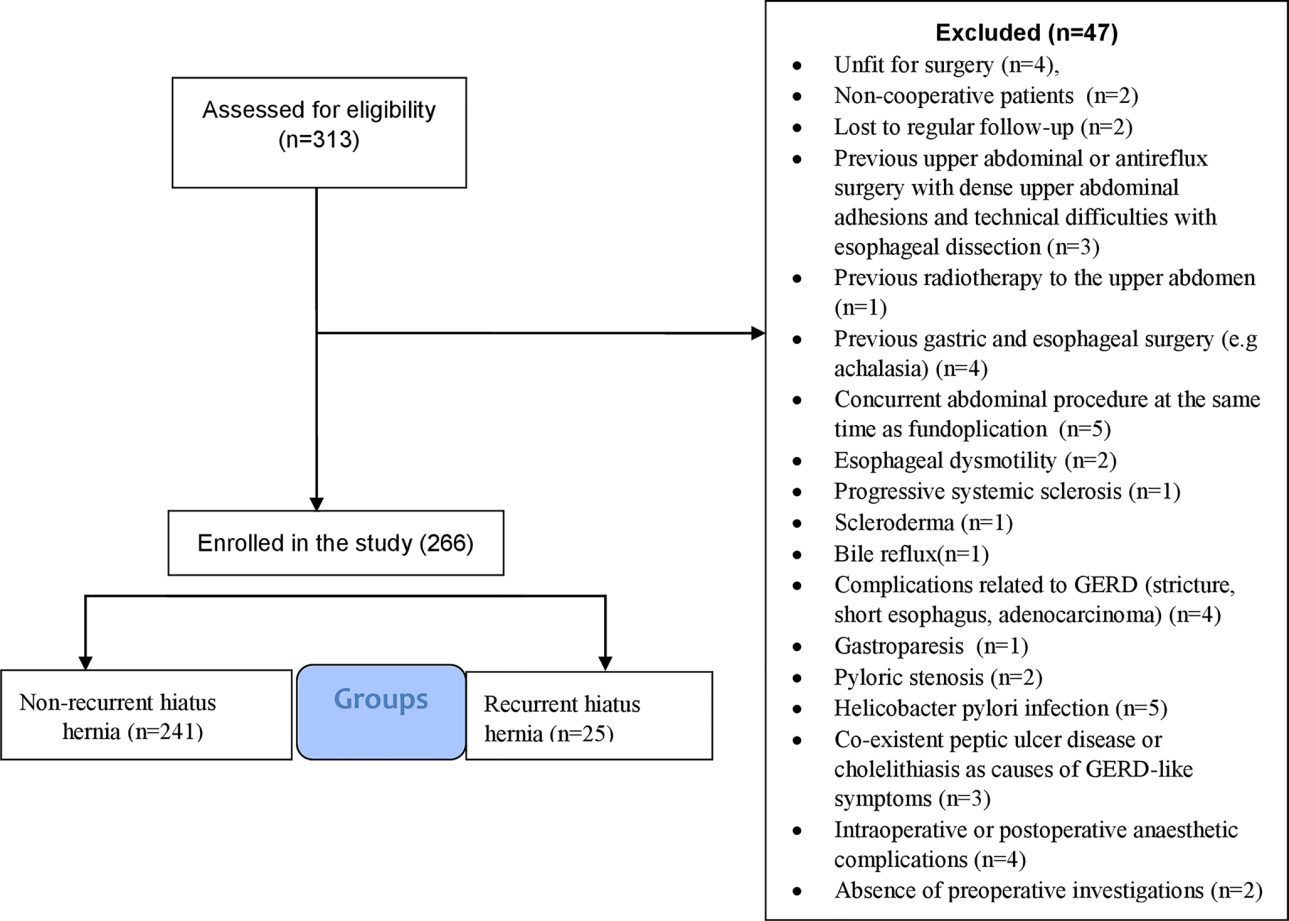
Flow chart of inclusion and exclusion criteria

This retrospective multicenter study evaluated 266 consecutive elderly patients aged ≥ 60 years [https://www.who.int/news-room/fact-sheets/detail/ageing-and-health]. The inclusion criteria included elderly patients who underwent LNF with *mesh cruroplasty* for giant (> 5 cm) [24] sliding HH with severe GERD (DeMeester score > 100) and refractory symptoms despite optimal medical therapy and were treated between March 2016 and March 2022. Patients were stratified into two groups based on HH recurrence: non-recurrent (*n* = 241), and recurrent (*n* = 25). The inclusion criteria were formulated according to the Society of American Gastrointestinal and Endoscopic Surgeons (SAGES) guidelines for GERD surgery for type I HH [25, 26]. The inclusion and exclusion criteria for the study participants are shown in Fig. 1. All participants exhibited a hypotensive lower oesophagal sphincter (LES) on high-resolution manometry (HRM) and abnormal pH impedance testing (DeMeester score > 100). Furthermore, the patients were surgically fitted, and informed consent was obtained prior to the procedure. The study participants completed a standardized three-year follow-up period. The study protocol was approved by the Institutional Ethics Committee and conducted in accordance with the principles of the Declaration of Helsinki, with registration in a clinical trial (NCT06935617).

**Fig. 2:**
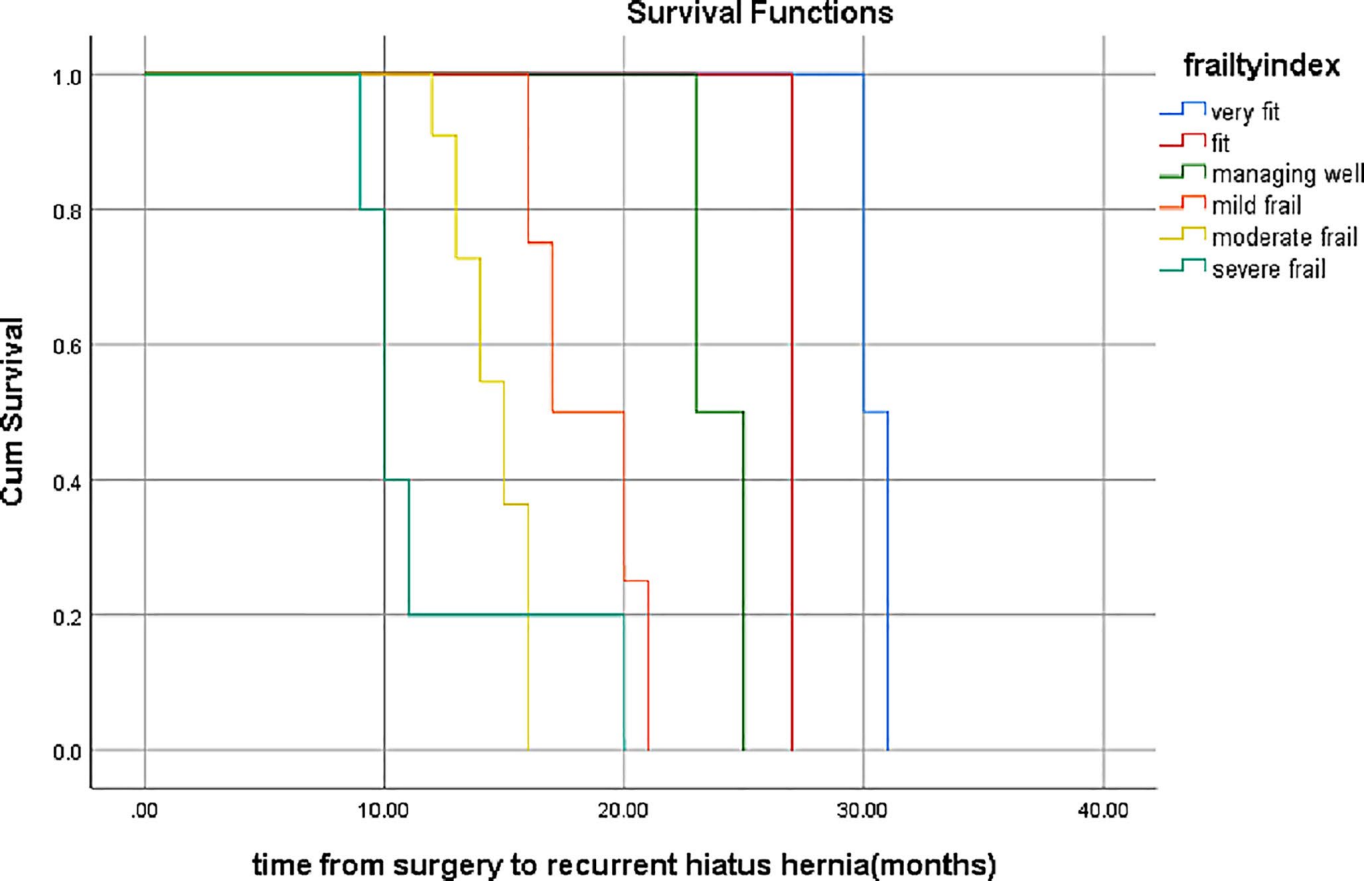
KM curve of recurrence free survival between frailty severity

#### Site recruitment

The patient cohort was drawn from four Egyptian medical institutions comprising academic referral centers (Zagazig University Hospital and Al-Azhar University Hospital), and two teaching hospitals (Mataryia Teaching Hospital and El Mahala Institute).

### Outcomes definition and measurement

The primary outcome was the incidence of postoperative recurrent sliding HH confirmed via clinical symptoms, barium swallow, endoscopy, or high-resolution manometry (HRM). Recurrence was defined as proximal displacement of the Z-line and gastric folds ≥ 2 cm above the diaphragmatic hiatus. The term ‘giant or large HH’ was defined as HH > 5 cm, with intraoperative crural defect size measured using sterile tape or calibrated graspers [27]. The secondary outcome was the association between frailty and HH recurrence assessed using the Clinical Frailty Scale (CFS).

### Diagnostic and assessment criteria

The modified DeMeester scoring system evaluates typical symptoms, including heartburn (HB), regurgitation, and dysphagia [28]. GERD severity was evaluated using the modified DeMeester score (DMS). DMS < 14.72: No GERD. DMS 14.72–50: Mild GERD. DMS 51–100: Moderate GERD. DMS > 100: Severe GERD. Testing was performed after discontinuation of acid-suppressive therapy. HRM was conducted to assess the LES pressure and configuration [29]. The severity of esophagitis was graded using the Los Angeles classification [30]. Failed fundoplication was classified according to Horgan criteria [31]. Intraoperative endomanometric evaluation of LES pressure and configuration was performed as previously described [32]. The Clinical Frailty Scale (CFS) was used to evaluate frailty as it is a validated tool for assessing functional decline in elderly surgical patients [33].

### Variables

Age, gender, smoking, diabetes mellitus (DM), coronary heart diseases (CHD), hypertension (HT), body mass index (BMI), ASA, Charlson comorbidity index, Frailty index, Times with symptoms before surgery (months), Response to acid reducing medications before surgery, Haitus hernia size cm, Preoperative initial symptoms, Modified demeester score of severity of initial symptoms, Preoperative esophagitis grading, Preoperative lower esophageal sphincter manometry (mmhg), Preoperative DeMeester score, Operative time (min), use of intraoperative endomanometry, Intraoperative esophageal sphincter manometry (mmhg), time taken to perform intraoperative manometry(min), time taken to perform intraoperative endoscope(min), Intraoperative complications, Endomanometry related complications, Dealing with intraoperative complications, Postoperative length of hospital stay, Early Postoperative complications, Clavien-Dindo (CD) classification, readmission within 30days, Dealing with early postoperative complications, 30 days mortality, Causes of mortality, Size of recurrent hernia (cm), time from surgery to recurrence(months)(median, IQR), Clinical Forms of recurrence, Treatment of recurrent HH, Causes of recurrence detected during reoperation, surgery for recurrent HH, Postoperative recurrent symptoms, Types of postoperative recurrent symptoms, Severity of recurrent GERD, DeMeester score of severity of recurrent symptoms, Appearance of new postoperative symptoms, Postoperative dysphagia score, Postoperative esophagitis grading, Lower esophageal manometry at the end of the study.

### Operative technique

All patients received Premedication: Oral flunitrazepam (1 mg), Induction: Etomidate (0.3 mg/kg) + fentanyl (2 µg/kg), Maintenance: Isoflurane (1–1.5 MAC) + fentanyl bolus (1 µg/kg/h), and neuromuscular blockade: Atracurium (0.5 mg/kg). *All patients underwent hiatal hernia mesh cruroplasty with concomitant laparoscopic Nissen fundoplication (LNF)*,* which is a well-established approach for managing giant sliding hiatal hernias (HH) with severe GERD in our centers. LNF is routinely performed alongside hiatal closure and mesh fixation in patients with HH to address both anatomical (hernia reduction*,* crural repair) and functional (anti-reflux) components*. Operative steps were performed as previously described [24, 32, 34]. *Dissection of the diaphragmatic hiatus to detect giant hiatal defect* [Supplementary Fig. 1]*. Mediastinal dissection was completed to obtain at least 3 cm of intraabdominal esophagus without tension.* Posterior hiatoplasty was done by approximation of the diaphragmatic crura posterior to the esophagus using 3–4 interrupted non-absorbable braided polyester sutures (Ethibond Excel, Ethicon Inc., Somerville, NJ, USA) [Supplementary Fig. 2]. The sutures were deeply placed, including the subdiaphragmatic fascia, immediate sequential suture tying to prevent fouling, and objective hiatal calibration. When difficult crural defects are closed with poor tissue quality characterized by attenuated muscular fibres and minimal fascial components, a primary tension-free crural approximation is often unachievable. To facilitate medial crural mobilization, we made right-sided lateral relaxing incisions. In order to avoid postoperative dysphagia, the cura was closed leaving almost 5 mm between the apex of the cruroplasty and the posterior wall of the esophagus. After crural repair, the diaphragmatic defects were reinforced with a U-shaped fenestrated *polypropylene-based composite mesh* (Ventralight™ ST Mesh, C.R. Bard, Inc.; Product #5954113) secured to the approximated crura using interrupted non-absorbable polypropylene sutures (2 − 0), or titanium helical tackers [Supplementary Fig. 3]. A 360° Nissen fundoplication was then constructed using 2–3 interrupted non-absorbable sutures (Ethibond™ 2 − 0, Ethicon) to create a 2-cm posterior wrap. Before fundoplication, oesophagal calibration was performed using a 52-French bougie to ensure the appropriate wrap dimensions. After LNF wrap formation, intraoperative HRM and endoscopy were performed only in the patients with endomanometric LNF. Postoperative admission to the intensive care unit (ICU) was performed when clinically indicated. All patients underwent transthoracic echocardiography within 6 h postoperatively to evaluate pericardial effusion, which was performed by certified cardiac sonographers.

#### Postoperative discharge and follow-up protocol

Patients were eligible for discharge when they met all of the following criteria: an asymptomatic status with stable vital signs, successful tolerance of liquid nutrition, and absence of symptoms (fever > 38 °C, heart rate > 100 bpm, respiratory rate > 20/min). All patients underwent a gastrografin contrast study on postoperative day 1 to assess possible esophageal perforation and evaluate repair integrity. The antiemetic protocol included Ondansetron 4–8 mg intravenous every 8 h for 24 h. Antitussive therapy included dextromethorphan 30 mg, as needed, every 6 h and activity restrictions. No heavy lifting was performed for six weeks. Scheduled evaluations were performed at 2 weeks (wound assessment), 6 weeks (diet progression clearance), and 6, 12, 24, and 36 months postoperatively. Annual endoscopic surveillance began at 12 months postoperatively. The multiple follow-up modalities included in-person clinic visits, telephone consultations, and electronic communication. Patient education regarding expected transient dysphagia (typically resolves in three months), potential new symptoms (gas-bloat syndrome), and delayed improvement of extraesophageal symptoms. Recurrence Surveillance: Symptomatic patients underwent a comprehensive evaluation, including Esophagogastroduodenoscopy, high-resolution oesophagal manometry, and contrast-enhanced esophagography.

#### Statistical analysis

Data analyses were conducted using IBM SPSS Statistics (version 28). We began by assessing the distribution of continuous variables using the Kolmogorov-Smirnov normality test and visual inspection of graphical plots. Continuous data are presented as mean ± standard deviation or median with interquartile range. We used the Student’s t-test for parametric data or the Mann-Whitney U test for nonparametric comparisons. Categorical variables were analyzed using the χ² test or Fisher’s exact test. To identify independent predictors of HH recurrence, we developed multivariable regression models. *Only variables with a p-value < 0.25 in the univariate analysis were included in the multivariable analysis.* We performed Kaplan-Meier survival analyses for time-dependent outcomes, particularly recurrence-free survival and the relationship between frailty and recurrence timing. We compared survival curves between groups using log-rank tests. Throughout all analyses, we maintained a consistent significance threshold of *p* < 0.05 (two-tailed) to determine statistical significance.

## Results

### Patient characteristics and preoperative data

Patient Characteristics and Preoperative Data are summarized in Table 1. Our study included 266 patients who underwent LNF, with 241 (90.6%) maintaining a successful repair and 25 (9.4%) developing recurrent HH. The baseline characteristics were similar between the groups, except for frailty status (*p* = 0.02) and duration of preoperative symptoms (*p* = 0.03), which showed statistically significant differences. Clinical data before surgery showed no significant differences in mean age (66.92 ± 4.3vs 67.79 ± 3.7 years, *p* = 0.25); the majority of patients in both groups were female (72.6% vs. 80%, *p* = 0.4), The mean BMI (31.36 ± 3.4 vs. 31.7 ± 3.9, *p* = 0.62), HB (73.4% vs. 68%, *p* = 0.4), regurgitation (20.8% vs. 32%, *p* = 0.3), hernia size (6.5 ± 0.8 cm vs. 6.4 ± 0.7, *p* = 0.31), mean Charlson comorbidity index (6.5 ± 1.7 vs. 6.7 ± 2.2, *p* = 0.58) and, grade C esophagitis (63.9% vs. 68%, *p* = 0.4) in the non-recurrence and the recurrence group, respectively. The frailty index was significantly associated with recurrence, with a higher proportion of patients with moderate and severe frailty in the recurrence group (44% vs. 17% and 20% vs. 13.3%, respectively; *P* = 0.02). Most preoperative initial symptoms were heartburn (HB) (73.4% vs. 68%, *p* = 0.2), with grade III HB being the most common Modified DeMeester score in both groups (51.5% vs. 48%). The median Preoperative DeMeester scores were 114(107–122) and 110(105–120) (*p* = 0.23) in the two groups, respectively.

**Table 1:** Patient characteristics and preoperative data between no recurrent hiatus hernia and recurrent hiatus hernia patients

variables		No recurrence (No = 241)	Recurrence (No = 25)	*p*-value
Age(years)(Mean ± SD)		66.92 ± 4.3	67.79 ± 3.7	0.25
Sex	male	66(27.4%)	5(20%)	0.4
	female	175(72.6%)	20(80%)	
Smoking		61(25.3%)	4(16%)	0.3
CHD		37(15.4%)	5(20%)	0.5
HTN		34(14%)	6(24%)6(24%)	0.3
DM		40(16.6%)	5(20%)	0.6
BMI (Mean ± SD)		31.36 ± 3.4	31.7 ± 3.9	0.62
ASA	ASA-I	165(68.5%)	16(64%)	0.8
	ASA-II	57(23.7%)	7(28%)	
	ASA-III	19(7.8%)	2(8%)	
Charlson comorbidity index (Mean ± SD)		6.5 ± 1.7	6.7 ± 2.2	0.58
Frailty index	Very fit	31(12.9%)	2(8%)	0.02*
	Fit	15(6.2%)	1(4%)	
	Managing well	47(19.5%)	2(8%)	
	mild frail	75(31.1%)	4(16%)	
	moderate frail	41(17%)	11(44%)	
	severe frail	32(13.3%)	5(20%)	
Times with symptoms before surgery (months)(Median, IQR)		23(15–33)	19(15–26)	0.03*
Response to acid-reducing medications before surgery		180(74.7%)	17(68%)	0.4
Haitus hernia size (cm)(Mean ± SD)		6.52 ± 0.8	6.4 ± 0.7	0.31
Preoperative initial symptoms	Atypical symptoms	14(5.8%)	0(0.00%)	0.2
	Heartburn	177(73.4%)	17(68%)	
	Regurgitation	50(20.8%)	8(32%)	
Modified Demeester score of severity of initial symptoms	No score	15(6.2%)	0(0.00%)	0.6
	Score 1 HB	24(10%)	1(4%)	
	Score 2 HB	29(12%)	4(16%)	
	Score 3 HB	124(51.5%)	12(48%)	
	Score 1 R	6(2.5%)	1(4%)	
	Score 2 R	14(5.8%)	2(8%)	
	Score 3 R	29(12%)	5(20%)	
Preoperative esophagitis grading	Grade A esophagitis	11(4.6%)	0(0.00%)	0.4
	Grade B esophagitis	15(6.2%)	3(12%)	
	Grade C esophagitis	154(63.9%)	17(68%)	
	Grade D esophagitis	61(25.3%)	5(20%)	
Preoperative lower esophageal sphincter manometry (mmHg)(Median, IQR)		6(5–8)	7(5–8)	0.468
Preoperative Demeester score (Median, IQR)	a-Total time pH less than four	64(56–72)	62(57–65)	0.32
	b-Time pH less than four upright	50(45–61)	50(43–58)	0.67
	c-Time pH less than four supine	34(33–41)	43(27–41)	0.31
	D-Number of reflux episodes per hour	17(13–20)	17(14–20)	0.4
	e-Number of reflux episodes more than five min	9(6–12)	11(8–16)	0.07
	f-Duration of longest episode in minutes	23(19–31)	21(18–28)	0.31
	g-Score value	114(107–122)	110(105–120)	0.23

IQR: Interquartile range; CHD: Coronary Heart Disease; HTN: Hypertension; HB: heartburn; R: Regurgitation; ASA: American Society of Anesthesiologist; CHD: coronary heart disease; DM: diabetes mellitus; BMI: body mass index; HTN: hypertension

*statistically significant

### Intraoperative and postoperative findings are presented in Table 2

**Table 2:** Intraoperative and postoperative findings of no recurrent hiatus hernia and recurrent hiatus hernia patients

		No recurrence (No=241)	Recurrence No(25)	*P*-value
Operative time (min)		57(55-70)	71(57-110)	0.009*
Use of intraoperative endomanometry		115(47.7%)	5(20%)	0.008*
Intraoperative esophageal sphincter manometry (mmHg)(Mean±SD)		32.45±4.6	35.80±5.2	0.11
Time taken to perform intraoperative manometry(min)(Median, IQR)		20(16-24)	18(15-23)	0.42
Time taken to perform intraoperative endoscope(min)(Median, IQR)		22(18-26)	18(16-23)	0.323
Intraoperative complications	No intraoperative complications	236(98%)	25(100%)	0.7
	Endomanometric related complications	0(0.00%)	3(12%)	
	Bleeding from short gastric arteries	2(0.8%)	0(0.00%)	
Endomanometry related complications	Esophageal wall bleeding	0(0.00%)	2(8%)	0.9
	Epistaxis	0(0.00%)	1(4%)	
Dealing with intraoperative complications	No treatment	236(98%)	25(100%)	0.9
	Argon beam coagulation	0(0.00%)	2(8%)	
	Control bleeding from short gastric arteries laparoscopically	2(0.8%)	0(0.00%)	
	Control epistaxis by adrenaline nasal pack	0(0.00%)	1(4%)	
Postoperative length of hospital stay (days)(Mean±SD)		2.4±0.5	2.7±0.5	0.01*
Early Postoperative complications	No postoperative complications	232(96.4%)	22(88%)	<0.001*
	Superficial SSI	1(0.4%)	1(4%)	
	Organ/space SSI	0(0.00%)	2(8%)	
	Ileus	1(0.4%)	0(0.00%)	
	Acute myocardial infarction	2(0.8%)	0(0.00%)	
	Pulmonary complications (infection/atelectasis)	3(1.2%)	0(0.00%)	
	Intra-abdominal collection	2(0.8%)	0(0.00%)	
CD classification	Grade 0	232(96.4%)	22(88%)	0.1
	Grade I	1(0.4%)	1(4%)	
	Grade II	1(0.4%)	0(0.00%)	
	Grade III	7(2.8%)	2(8%)	
Readmission within 30days	No early readmission	232(96.4%)	22(88%)	0.058
	Early readmission	9(3.6%)	3(12%)	
Dealing with early postoperative complications	Open wound in bed	1(0.4%)	1(4%)	0.053
	Conservative treatment for ileus	1(0.4%)	0(0.00%)	
	ICU admission with cardiorespiratory support	5(2%)	0(0.00%)	
	Sonar guided aspiration for intra-abdominal collection	2(0.8%)	0(0.00%)	
	Reoperation for organ/space SSI	0(0.00%)	2(8%)	
30 days mortality	No mortality	235(97.5%)	24(96%)	0.6
	Mortality	6(2.5%)	1(4%)	
Causes of mortality	Septic shock	1(0.4%)	1(4%)	0.08
	Cardiorespiratory failure	5(2%)	0(0.00%)	

CD: classification: Clavien-Dindo, IQR: Interquartile range

*statistically significant

All procedures were completed laparoscopically without major complications. The median operative time was significantly longer in the recurrence group (71 [57–110] min vs. 57 [55–70] min; *p* = 0.009). The use of intraoperative manometry differed significantly between groups (47.7% vs. 20%, *p* = 0.008). Three patients (1.2%) in the recurrence group experienced HRM/endoscopy-related complications: esophageal wall bleeding (*n* = 2, 8%) managed with argon plasma coagulation and epistaxis (*n* = 1, 4%) controlled with nasal packing. The mean duration of HRM (32.5 ± 4.6 vs. 35.8 ± 5.2 min, *p* = 0.11) and endoscopy (20 [16–24] vs. 18 [15–23] min, *p* = 0.42) did not differ significantly between groups. The mean postoperative hospital stay was significantly longer in the recurrent group (2.4 ± 0.5 vs. 2.7 ± 0.5 days, *p* = 0.01). Superficial surgical site infections (0.4% (*n* = 1) vs. 4% (*n* = 1), *p* = 0.2), organ/space infections (8% (*n* = 2) vs. 0%), and 30-day mortality rates (2.5% (*n* = 6) vs. 4% (*n* = 1), *p* = 0.6) were observed in the recurrent and nonrecurrent groups, respectively.

### Primary outcome (Table 3)

**Table 3:** Primary outcome of no recurrent hiatus hernia and recurrent hiatus hernia patients

Variables		No recurrence (No=241)	Recurrence No(25)	*P* value
Size of recurrent hernia (cm) (median, IQR)			5.2(4.1-6)	
Time from surgery to recurrence (months) (median, IQR)			16(13-20)	
Clinical Forms of recurrence	Symptomatic recurrence		22(88%)	
	Asymptomatic recurrence (radiological)		3(12%)	
Treatment of recurrent HH	reoperation		12(48%)	
	The patient refused reoperation(the causes of recurrence were not detected)		13(52%)	
Causes of recurrence detected during reoperation	Type I herniated fundoplication(GEJ herniated through the hiatus, with the fundoplication-type IA)		1(4%)	
	Inadequate closure of the diaphragmatic crura		4(16%)	
	Incomplete sac excision		1(4%)	
	Crural sutures breakdown		5(20%)	
	Mesh disruption		1(4%)	
Surgery for recurrent HH	Laparoscopic redo surgery		8(32%)	
	Open abdominal redo surgery		4(16%)	
Postoperative recurrent symptoms	No	239(99.2)	3(12%)	
	Yes	2(0.8%)	22(88%)	<0.001*
Types of postoperative recurrent symptoms	No recurrent symptoms	239(99.2)	3(12%)	<0.001*
	Recurrent HB	2(0.8%)	14(56%)	
	Recurrent R	0(0.00%)	8(32%)	
The severity of recurrent GERD	No GERD	239(99.2)	3(12%)	<0.001*
	Mild GERD	2(0.8%)	10(40%)	
	Severe GERD	0(0.00%)	12(48%)	
Demeester score of severity of recurrent symptoms	Score 0(HB, R)	239(99.2%)	3(12%)	<0.001*
	Score 1 HB	0(0.00%)	8(32%)	
	Score 2 HB	1(0.4%)	2(8%)	
	Score 3 HB	0(0.00%)	5(20%)	
	Score 1 R	1(0.4%)	2(8%)	
	Score 2 R	0(0.00%)	1(4%)	
	Score 3 R	0(0.00%)	4(16%)	
Appearance of new postoperative symptoms	Dysphagia	11(4.6%)	0(0.00%)	<0.001*
	Gas bloat syndrome	4(1.6%)	0(0.00%)	
Postoperative dysphagia score	Score 1	2(0.8%)	0(0.00%)	<0.001*
	Score 2	4(1.6%)	0(0.00%)	
	Score 3	5(2.1%)	0(0.00%)	
Postoperative esophagitis grading	No esophagitis	239(99.2)	3(12%)	<0.001*
	Grade A esophagitis	1(0.4%)	5(20%)	
	Grade B esophagitis	1(0.4%)	5(20%)	
	Grade C esophagitis	0(0.00%)	7(28%)	
	Grade D esophagitis	0(0.00%)	5(20%)	
Lower esophageal manometry at the end of the study (median, IQR)		15(15-19)	15(15-16)	0.002*
Postoperative DeMeester score (at the end of the study) (median, IQR)	a-Total time pH less than four	3(2-4)	23(12-67)	<0.001*
	b-Time pH less than four upright	3(2-4)	12(10-55)	<0.001*
	C-Time pH less than four supine	3(2-4)	16(7-34)	<0.001*
	d- Number of reflux episodes per hour	3(2-4)	16(8-17)	<0.001*
	e- Number of reflux episodes more than five minutes	3(2-4)	10(6-14)	<0.001*
	f- Duration of longest episode in minutes	3(2-4)	21(9-33)	<0.001*
	g-Score value	12(10-13)	47(43-151)	<0.001*

IQR: Interquartile range; HH: Hiatus Hernia; GERD: Gastroesophageal Reflux Disease; HB: heartburn; R: Regurgitation; GEJ: Gastroesophageal Junction

*statistically significant

**Table 4:** Univariate and multivariate logistic regression analysis to predict postoperative recurrent hiatus hernia

	Univariate		Multivariate	
	OR (95% CI)	*p*-value	OR (95% CI)	*p*-value
Age (years)	1.065(0.957–1.186)	0.24	1.1 (0.9–1.2)	0.116
Sex	1.5(0.544–4.18)	0.43	-	-
Hiatus hernia size	0.746(0.423–1.315)	0.3	-	-
**Frailty index**	**1.4(1.04–1.95)**	0.02*	**1.4(1.003–1.982)**	0.04*
**Postoperative length of hospital stay**	**2.7(1.1–6.3)**	0.01*	**3.6(1.4–9.1)**	0.005*
Smoker	1.77(0.588–5.38)	0.3	-	**-**
Times with symptoms before surgery (months)	0.973(0.940–1.005)	0.09	0.9(0.9-1.004)	0.08
Preoperative esophagitis grading	0.96(0.53–1.73)	0.892		
Preoperative lower esophageal manometry pressure	1.068(0.899–1.268)	0.455	-	-
Operative time (min)	0.976(0.957–0.994)	0.01*	0.98(0.909–1.057)	0.6
BMI > 30	3.6(1.3-10.04)	0.01*	1.3(0.02–86.38)	0. 8
Use of intraoperative endomanometry	1.03(0.915–1.159)	0.62	-	**-**
Early Postoperative complications	1.05(0.673–1.63)	0.8	-	**-**

*Significant p-value; OR: Odds ratio; 95% CI: 95% confidence interval

The median size of the recurrent HH was 5.2 cm (IQR 4.1-6.0), with recurrence occurring at a median of 16 months (IQR 13–20) postoperatively. While most recurrences were symptomatic (88%(*n* = 22]), we identified three asymptomatic patients (12%) through routine imaging. Nearly half of the recurrent cases (*n* = 12, 48%) required reoperation primarily through laparoscopic approaches (*n* = 8, 32%). Intraoperative findings during revision surgery revealed several failure mechanisms, including crural closure failure (*n* = 4, 16%), suture breakdown (*n* = 5, 20%), mesh disruption (*n* = 1, 4%), and type IA herniated fundoplication (*n* = 1, 4%). Postoperatively, recurrent GERD symptoms were significantly more prevalent in the recurrence group (88% vs. 0.8%, *p* < 0.001), with HB (*n* = 14, 56%) and regurgitation (*n* = 8, 32%) being the most common. The objective assessment revealed severe GERD (DeMeester score > 100) in 48% (*n* = 12) of recurrent patients. High-resolution manometry demonstrated lower LES pressures in the recurrence group (15–16 mmHg vs. 15–19 mmHg, *p* = 0.02), while 24-hour pH monitoring showed significantly greater acid exposure (total time pH < 4:23 vs. 3, *p* < 0.001; supine reflux: 16 vs. 3, *p* < 0.001). Endoscopic evaluation revealed higher grades of esophagitis (LA classification grade C/D, 48%) in the recurrent patients.

### Predictors of recurrence HH

Multivariate analysis confirmed that the frailty index (odds ratio [OR], 1.4; 95% CI, 1.003–1.982, *p* = 0.04) and postoperative length of hospital stay (OR, 3.6; 95% CI, 1.4–9.1, *p* = 0.005) were independent risk factors for recurrence (Table 4).

### The relationship between frailty index and recurrent hiatus hernia (Fig. 2, Table 5”A-B”)

**Table 5A:** Relations of frailty index with recurrence

Frailty index		time	Estimate	Number of cumulative events	Number of remaining cases
Very fit	1	30.000	.500	1	1
	2	31.000	.000	2	0
Fit	1	27.000	.000	1	0
Managing well	1	23.000	.500	1	1
	2	25.000	.000	2	0
mild frail	1	16.000	.750	1	3
	2	17.000	.500	2	2
	3	20.000	.250	3	1
	4	21.000	.000	4	0
moderate frail	1	12.000	.909	1	10
	2	13.000	.	2	9
	3	13.000	.727	3	8
	4	14.000	.	4	7
	5	14.000	.545	5	6
	6	15.000	.	6	5
	7	15.000	.364	7	4
	8	16.000	.	8	3
	9	16.000	.	9	2
	10	16.000	.	10	1
	11	16.000	.000	11	0
severe frail	1	9.000	.800	1	4
	2	10.000	.	2	3
	3	10.000	.400	3	2
	4	11.000	.200	4	1
	5	20.000	.000	5	0

Test of equality of survival distributions for the different levels of frailty index

**Table 5B:** Overall comparisons

	Chi-Square	df	Sig.
Log Rank (Mantel-Cox)	22.954	5	.000
Breslow (Generalized Wilcoxon)	20.702	5	.001
Tarone-Ware	21.558	5	.001

Test of equality of survival distributions for the different levels of frailty index

1. Association between Frailty Index and Recurrence: Kaplan-Meier analysis (Fig. 2) revealed a significant divergence in recurrence-free survival based on frailty severity (log-rank test, *p* < 0.001). Very fit, fit, and managing well were associated with the lowest recurrence rates (≤ 50% at 23–30 months). Patients with mild frailty showed a recurrence rate of 75% after 16 months. Moderate-to-severely frail patients had the poorest outcomes, with moderate frailty and 90.9% recurrence within 12 months. Severe frailty: 80% recurrence within 9 months.
2. Cumulative Recurrence Events by Frailty Level (Table 5).

- A dose-dependent relationship was observed: very fit, fit, and managing well, with only one cumulative event lasting for 30 months. Mild frailty: 3 events by 16 months (75% recurrence). Moderate frailty: 11 events by 16 months (100% recurrence). Severe frailty: 4 events within 9 months (80% recurrence).
- Statistical significance: All tests (log-rank, Breslow, and Tarone-Ware tests) confirmed frailty as a strong predictor (*p* < 0.001).

3. Time-to-recurrence pattern: Recurrence occurred earlier with increasing frailty (median time to recurrence: severe frailty, 9 months). Moderate frailty: 12 months. Mild frail: 16 months. Very fit, fit, and managing well: >23 months.

## Discussion

### 1. Summary of key findings and primary outcome

Our study addresses a critical knowledge gap by demonstrating that frailty independently predicts recurrence in EP with large HH (> 5 cm) and severe GERD (DeMeester > 100), showing a 9.4% recurrence rate, which compares favorably with published rates of 10.1–42% [35–38] and higher than other study [39]. Multivariate analysis confirmed frailty as a key determinant, with survival curves revealing that moderately/severely frail patients experienced 80–90% 1-year recurrence versus > 50% 2-year recurrence-free survival in fit patients, whereas the median time-to-recurrence decreased progressively from > 23 months to 9 months across frailty strata. The predominant failure mechanism, crural suture breakdown, aligns with prior reports [40]. Our reduced incidence of recurrent sliding HH was likely due to rigorous preoperative smoking cessation, aggressive antiemetic/antitussive protocols, and meticulous surgical techniques. Polypropylene-based composite mesh reinforcement (U-shaped configuration with a preserved esophageal cuff) provides additional durability benefits through circumferential tissue contact and overlap, which is consistent with biomechanical principles. Mesh-related complications can be avoided by careful tissue spacing [41–43]. These findings underscore the interplay between patient factors (particularly frailty) and technical precision in determining surgical outcomes in this complex population.

### **2**. Frailty index as a risk factor for recurrent HH

Our findings establish frailty as a critical, modifiable risk factor for recurrent HH, distinct from the traditional anatomical or technical considerations. A striking dose-dependent relationship was observed, where moderate/severe frailty predicted a high recurrence rate. Conventional paradigms have focused solely on BMI, hernia size, or transient stressors [16, 37, 44]. We recommend routine frailty screening for patients with giant HH. These results are consistent with those of a previous study linking frailty to poor surgical outcomes [45]. A gradual decrease in physiological reserve occurs with aging; however, this decrease is accelerated in frailty, and homoeostatic mechanisms begin to fail [46]. Therefore, an important aspect of frailty is how the complex mechanisms of aging promote a cumulative decline in several physiological systems, subsequent depletion of homoeostatic reserves, and vulnerability to disproportionate changes in health status after minor stressor events [47].

Frailty arises from cumulative dysfunction across the neurological, endocrine, immune, and musculoskeletal systems, impairing stress response and tissue repair. Neurologically, frailty-associated hippocampal dysfunction promotes chronic glucocorticoid excess [48], inducing diaphragmatic sarcopenia and connective tissue catabolism, which in turn promotes muscle catabolism (sarcopenia), weakens the diaphragmatic crura and LES, and increases the risk of HH recurrence. Dysautonomia may disrupt gastroesophageal motility and exacerbate gastroesophageal reflux disease (GERD) and mechanical stress during crural repair. Neuroinflammatory mediators (elevated IL-6/TNF-α) increase in frail persons [49] and impair wound healing through matrix metalloproteinase activation, which impairs postoperative healing of the fundoplication site and reduces diaphragmatic strength. Additionally, delirium risk compromises postoperative compliance with postoperative restrictions (e.g., lifting, bending), aspiration risk due to impaired swallowing coordination, prolonged immobilization, delayed recovery, stress of repair and modifications (e.g., avoiding large meals), medication regimens (e.g., PPIs and prokinetics), and lifestyle restrictions (e.g., weight management and smoking cessation) [50]. Immunologically, the pro-inflammatory mediator associated with frailty generates glycation endproducts that disrupt crural collagen integrity [51]. Muscularly, sarcopenia is defined as the progressive loss of skeletal muscle mass, strength, and power and is regarded as a key component of frailty [52]. Loss of muscle strength and power may be more important than changes in muscle mass [53]. Under normal circumstances, muscle homeostasis is maintained through a delicate balance between new muscle cell formation, hypertrophy, and protein loss. This balance is coordinated by the brain, endocrine system, and immune system and is affected by nutritional factors and the amount of physical activity. The adverse neurological, endocrine, and immune components of frailty have the potential to disrupt this delicate homoeostatic balance and accelerate the development of sarcopenia. Loss of muscle mass and strength compromises crural integrity, reduces functional ability, and increases mechanical strain during repair. Our results align with evolving geroscience frameworks [45–46] while offering specific clinical applicability; frailty assessment better predicts vulnerability than chronological age or isolated risk factors.

#### Strength and limitation

The retrospective design inherently carries a risk of selection bias. We acknowledge that certain potentially influential factors, including variations in surgical technique among centres and preoperative medication use, could not be systematically analysed. However, we implemented standardised frailty assessments across all participating centres to provide objective measures this critical prognostic factor. Additionally, our rigorous follow-up protocol, with uniform outcome measures collected at predetermined intervals, helps to mitigate some limitations typical of retrospective analyses. While introducing some heterogeneity, our study’s multicenter nature enhances the generalizability of our findings to broader clinical practice. *Future studies should prospectively compare closure methods while stratifying by frailty and evaluate different hiatal closure methods as predictors for recurrence.*

## Conclusions

Frailty is a key determinant of surgical outcomes in patients with EP undergoing HH repair. Moderate-to-severe frailty independently predicted HH recurrence and substantially decreased recurrence-free survival. This compels us to reconsider how we evaluate surgical candidates, moving beyond calendar age, to assess physiological reserves using validated frailty measures. These results have important practical implications for clinical practice. First, routine frailty assessment could transform patient selection by identifying EP who stand to benefit from surgery despite advanced age while flagging high-risk patients who might need alternative approaches. Second, detecting frailty creates opportunities for targeted interventions, such as nutritional optimisation, physical rehabilitation, and medical management, which may improve surgical resilience. Third, these assessments provide a foundation for more meaningful shared decision-making by quantifying individualized risk profiles. Our study suggests that incorporating frailty metrics into standard preoperative workflows could enhance outcomes across multiple dimensions, including improved patient selection, optimised timing of intervention, and better long-term results. Prospective studies are needed to determine whether preoperative optimization of frail patients can reduce recurrence rates, a crucial question this study has helped bring into focus.

## Electronic supplementary material

Below is the link to the electronic supplementary material.

Supplementary Material 1

Supplementary Material 2

Supplementary Material 3

## Data Availability

The dataset(s) supporting the conclusions of this article is(are) confidential and will be available upon request to the corresponding author.
