# Supplementary figures and images for: Frailty predicts recurrence after laparoscopic Nissen fundoplication with mesh cruroplasty for giant sliding hiatal hernia with severe reflux esophagitis in elderly patients: a multicenter retrospective study
Source: Hernia. 2025 Jul 18;29(1):235. doi: 10.1007/s10029-025-03416-6 (PMC12274264; doi:10.1007/s10029-025-03416-6)

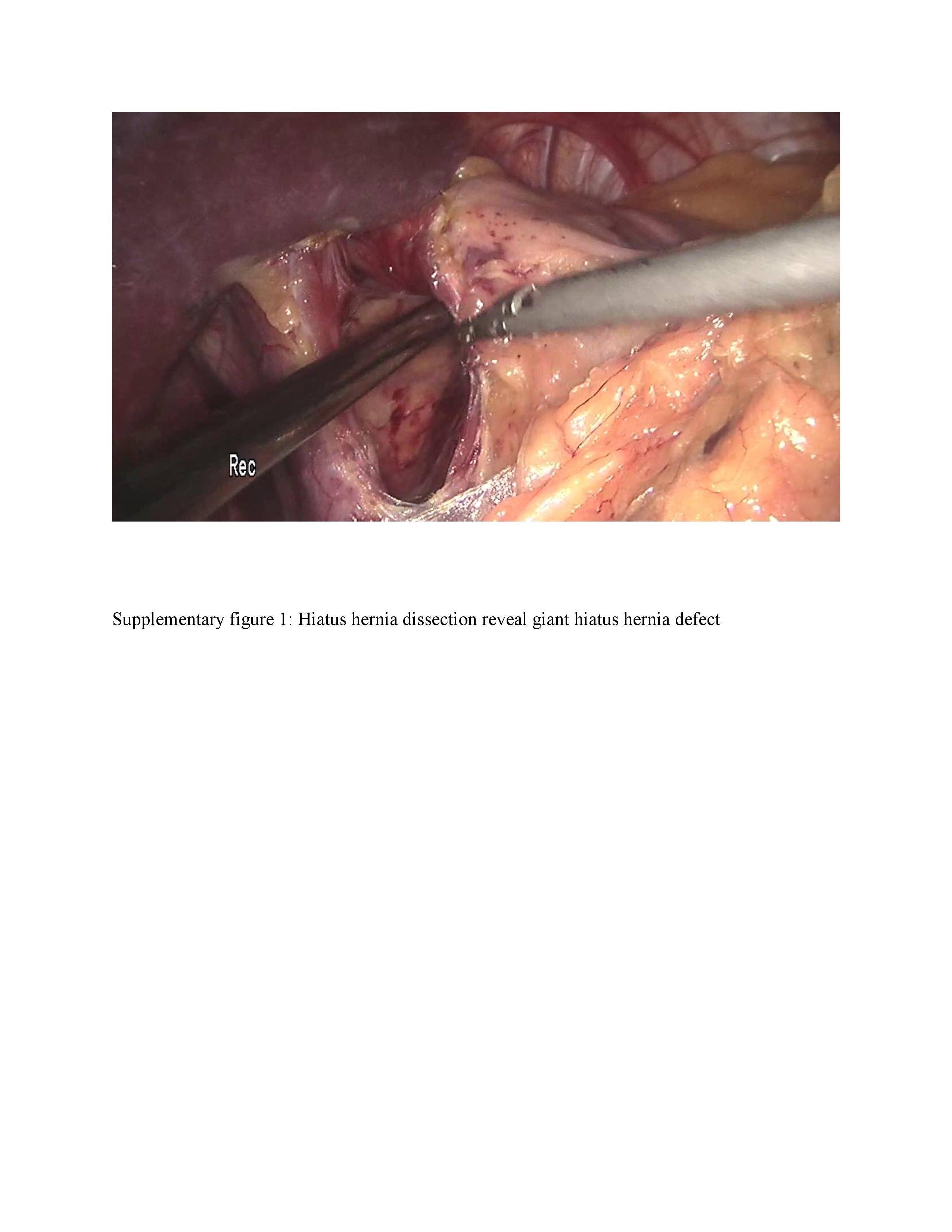

Supplement: Supplementary file 1 — Supplementary Material 1 [file 10029_2025_3416_MOESM1_ESM.docx]

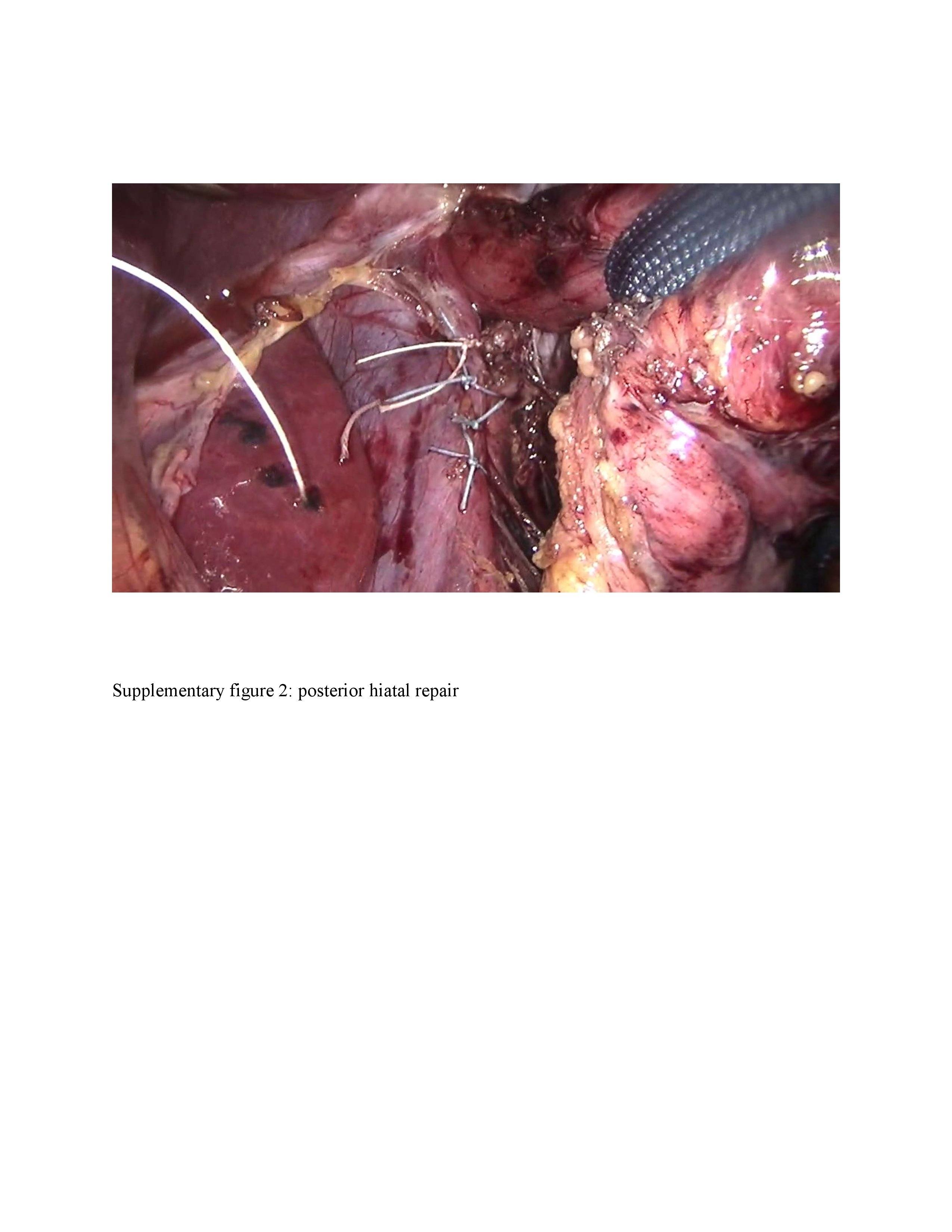

Supplement: Supplementary file 2 — Supplementary Material 2 [file 10029_2025_3416_MOESM2_ESM.docx]

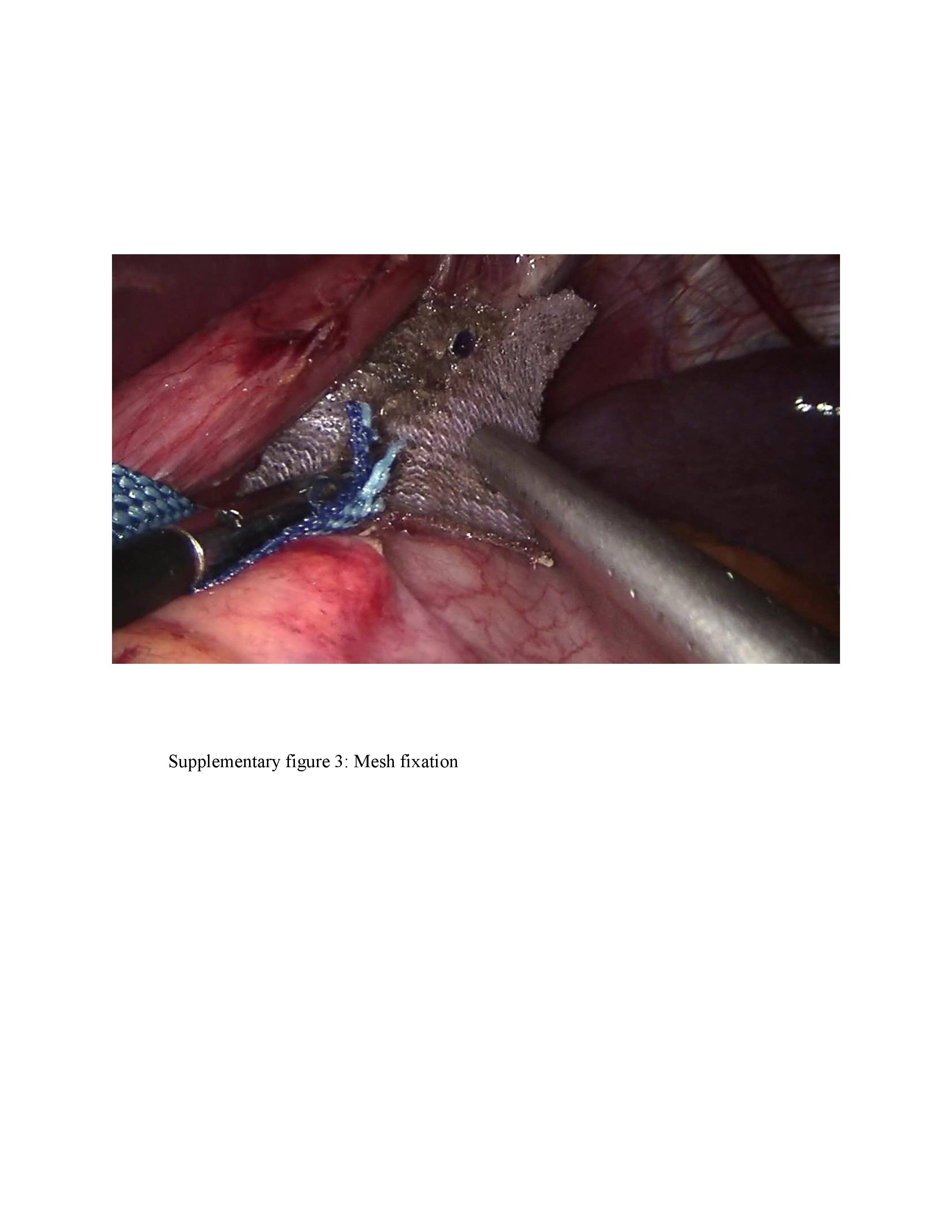

Supplement: Supplementary file 3 — Supplementary Material 3 [file 10029_2025_3416_MOESM3_ESM.docx]
